# Understanding unusual sensory experiences: a randomised experimental study of a school‐based intervention for adolescents

**DOI:** 10.1111/camh.12651

**Published:** 2023-03-23

**Authors:** Jerica Radez, Louise Johns, Felicity Waite

**Affiliations:** ^1^ Oxford Institute of Clinical Psychology Training and Research, Medical Sciences Division University of Oxford Oxford UK; ^2^ Oxford Health NHS Foundation Trust Oxford UK; ^3^ Department of Psychiatry University of Oxford Oxford UK

## Abstract

**Background:**

One in ten young people experience unusual sensory experiences (USE), such as hallucinations. From a cognitive perspective, the appraisal of USE determines the impact of these experiences. Negative appraisal, as well as other psychological processes (e.g. thinking flexibility, maladaptive schemas, anxiety/depression), is associated with more distress. Our aim was to (a) develop a universal single‐session school‐based intervention on USE for adolescents and (b) evaluate the effect of the intervention on appraisals of and help seeking intentions for USE.

**Methods:**

A randomised controlled experimental design with a one‐month follow‐up was used to test the effectiveness of the intervention in one school. Students (*n* = 223) aged 12–13 were randomised by class to a single‐session intervention on USE or a control intervention (generic mental wellbeing). Participants completed measures of appraisals of and help‐seeking intentions for USE at pre‐ and postintervention and at one‐month follow‐up. They also completed measures of schemas, thinking flexibility and anxiety/depression at preintervention.

**Results:**

Overall, 190 adolescents completed the main outcome measures at all three points. The intervention on USE led to a significant (*p* < .05) increase of positive appraisals of USE compared with the control, with effects sustained at one‐month follow‐up. The intervention on USE did not lead to significantly greater help‐seeking intentions for USE (*p* = .26). Adolescents' schemas were associated with appraisals and slow thinking and anxiety/depressive symptoms with help‐seeking behaviour for USE.

**Conclusions:**

A single‐session universal school‐based intervention shows promise by improving appraisals of USE. Further research is required across different school populations.

**Key Practitioner Message**

- Unusual sensory experiences (USE) are experienced by up to 15% of children and adolescents in the general population. Although usually transient, they can lead to high levels of distress and stigma for some young people.
- From a cognitive perspective, the way that young people make sense of USE (appraisal) is crucial in determining the distress and impact of these experiences. Psychoeducational interventions aiming to increase normalising and nonthreatening appraisals of USE for young people might reduce the negative impact of these experiences.
- We designed and evaluated a single‐session school‐based intervention for young people aged 12–13 years. We compared the intervention with a control condition (i.e. generic wellbeing intervention).
- Adolescents receiving the intervention on USE reported more positive appraisals of USE immediately after the intervention and at one‐month follow‐up. The intervention did not lead to changes in help‐seeking intentions for USE. In general, more adaptive schemas were associated with more positive appraisals of USE, whereas lower levels of anxiety/depressive symptoms and slower (i.e. rational) thinking were associated with higher intentions to seek help for USE.
- Results of this study suggest that a simple, single‐session psychoeducational intervention focused on adolescents' appraisals of USE has the potential to lead to positive and lasting changes in appraisals of USE.
- Future research should focus on developing and evaluating psychoeducational interventions that also target identified protective factors.

## Introduction

Between 10% and 15% of children and adolescents in the community report experiencing unusual sensory experiences (USE), such as visual and auditory hallucinations (Kelleher et al., 2012, 2015). In this study, USE are described as all situations where there is a discrepancy between what is perceived by a young person and what exists in the real world. We focused on multimodal sensory experiences, that is, auditory, visual, olfactory, gustatory and bodily sensations (e.g. Jardri et al., 2014), and other less well‐known sensory experiences, such as sensing the presence of another person. Although commonly associated with symptoms of serious mental health problems, such as psychosis, USE for young people usually spontaneously resolve (Bartels‐Velthuis, van de Willige, Jenner, van Os, & Wiersma, 2011). However, young people can find these experiences distressing (Parry, Loren, & Varese, 2021) and highly stigmatising (Bogen‐Johnston, de Visser, Strauss, Berry, & Hayward, 2019), which is associated with persistence of these experiences.

Cognitive models of USE and similar psychotic‐like experience (PLEs) emphasise the central role of appraisals (the way that people make sense of experiences) in determining the impact of USE and predicting the distress and subsequent need for professional help (Freeman, 2016; Garety, Kuipers, Fowler, Freeman, & Bebbington, 2001; Morrison, 2001). Research with adults suggests that personalising, distressing and threatening appraisals of USE lead to higher levels of impact and distress, whereas normalising and supernatural appraisals of USE tend to be associated with a more favourable outcome (Gaynor, Ward, Garety, & Peters, 2013; Peters et al., 2017; Ward et al., 2014). Similarly, recent studies with young people suggest that developing personal meaning‐making explanations of USE leads to lower levels of distress than understanding USE through the lens of a potential serious mental health problem (Parry & Varese, 2021).

There are many different psychological processes (henceforth referred to as ‘covariates’) that are associated with someone's appraisals of USE. One cognitive model argues that dysfunctional appraisals of USE are maintained by reasoning processes (e.g. belief inflexibility), maladaptive schemas (i.e. negative beliefs about oneself, other people, the world), adverse social environments, emotional processes (e.g. anxiety and depression) and secondary appraisal (e.g. stigma associated with mental health and help‐seeking) (Garety et al., 2001). Although there is no single accepted psychological model of USE in adolescents, research suggests that psychological processes identified in adult models, such as maladaptive schemas (Anilmis et al., 2015), reasoning processes (Hassanali et al., 2015) and internalising/externalising problems (Lancefield, Raudino, Downs, & Laurens, 2016), apply to young people as well. Understanding the role of the above processes in predicting the distress of USE is important as it can help us develop targeted interventions for reducing (a) the negative impact and (b) the stigma associated with USE for young people (Gin et al., 2021).

Psychoeducation focusing on normalising and destigmatising USE can be instrumental in reducing the overall distress and negative impact of USE for young people (Maijer et al., 2019; Parry & Varese, 2021). Given the high prevalence of USE in the adolescent general population, there is an opportunity to deliver psychoeducational interventions through educational settings (Parry, 1992; Parry & Varese, 2021). However, existing school‐based interventions mainly focus on common mental health problems, such as anxiety and depression (Fazel, Hoagwood, Stephan, & Ford, 2014), despite clinicians' and young people's preference for interventions with a transdiagnostic focus (Garralda, 2015; Kapur et al., 2014). The recently developed and evaluated CUES‐Ed is a universal school‐based intervention, focusing on promoting nonstigmatising appraisals of USE in primary age children. The intervention is delivered through eight school lessons and the preliminary results in preadolescent children are promising (Underwood et al., 2021). However, to our knowledge, no school‐based intervention has yet focused only on USE and been provided to adolescents.

In this randomised controlled experimental study, we aimed to develop and evaluate a single‐session school‐based intervention on USE in adolescents. The study had two primary and two secondary hypotheses. In our primary hypotheses, we predicted that, compared with a control condition, the intervention would (a) lead to an increase in positive (e.g. normalising) appraisals of USE in adolescents and (b) lead to an increase in young people's help‐seeking intentions regarding USE. We also hypothesised that these effects would be maintained over time (measured at one‐month follow‐up). Our secondary hypotheses predicted that the individual's flexibility of thinking, self‐perceptions/schemas and anxiety and depression symptoms would be significant covariates of the relationship between the intervention and (a) appraisals of USE and (b) help‐seeking intentions regarding USE.

## Method

The study was pre‐registered at the Open Science Framework (Radez, Waite, & Johns, 2022).

### Interventions

#### Understanding sensory experiences in adolescents (USE‐A)

The USE‐A is a single session (40 min) educational session delivered in school by a mental health practitioner with the aim of increasing adolescents' understanding of USE. The intervention consisted of the following topics: (a) definition of USE including examples of sensory experiences that are odd and unexpected (e.g. optical illusions, auditory hallucinations), and reasons for different sensory experiences (e.g. lack of sleep, drugs, illness, high anxiety, grief), (b) explanation using a cognitive model including examples of how different interpretations of sensory experiences influence the way we feel and act in response to them (e.g. how threatening interpretations of USE lead to higher levels of negative emotions and can increase an overall impact and distress of these experiences) and (c) help‐seeking for USE (i.e. examples of when and where to seek help for USE).

The intervention – based on the cognitive models of psychosis (e.g. Garety et al., 2001) – was developed by JR, FW and LJ and modified upon consulting additional researchers and clinicians working with young people. The contents of the intervention were also informed by treatment protocols for managing USE in adults (e.g. Dodgson et al., 2021). In addition, the researchers sought input from mental health charities and secondary school teachers. The final version of the intervention was piloted with young people aged 11–13 to ensure it was understandable and engaging for the target population.

#### Control intervention

A single session (40 min) educational intervention on general mental health and wellbeing topic was developed as an active control condition. The control intervention was entitled ‘Be physically active – 5 steps to mental wellbeing’, and this topic was selected by the participating school.

### Procedure

This study took place in a large (>1500 pupils) mixed state school in Southeast England. The school distributed parental study information leaflets and opt‐out forms electronically to all parents of Year 8 (12–13 years) students (*N* = 270) in April 2022. At the same time, the school also electronically distributed adolescent information leaflets to all Year 8 (12–13 years) students. After 2 weeks, a researcher delivered one of the interventions in person during one school lesson (60‐min). All classes (*n* = 10) were randomly assigned to either the experimental or control intervention, with class being used as a clustering variable. Prior to each intervention, adolescents were asked to fill in five brief questionnaires (see Measures), taking approximately 15 min in total. Following initial questionnaire completion, the intervention was delivered, which lasted up to 40 min. Adolescents were then asked to fill the Appraisals Measure and GHSQ immediately after the intervention. All the interventions were delivered within 2 weeks with nine delivered by the lead researcher (JR) and one by another researcher (LJ). For the follow‐up, adolescents were asked to fill in the Appraisals Measure and GHSQ after approximately 1 month. This was done using paper forms during the class tutor time. The lead researcher (JR) collected paper forms from the school as soon as they were completed. After data collection was completed, all young people in the control condition were given access to a pre‐recorded intervention on USE via e‐learning. No personal data were collected, and students used anonymised, unique ID codes to ensure that their follow‐up questionnaire responses were matched.

### Measures

The measures used to assess help‐seeking, anxiety and depression symptoms, and schemas have all been developed and evaluated with young people. However, there is a lack of validated measures of appraisals of USE and reasoning processes in young people. Therefore, we adapted the appraisals measure for ‘unusual experiences in children’ developed by Bradley et al. (2013). To ensure that the final set of outcome measures was appropriate to use with young people, we piloted the questionnaire measures with young people aged 11–13. All young people reported finding the measures appropriate and understandable.

#### Primary outcome measures

##### Measure of Appraisal of USE

We generated a 6‐item measure assessing appraisals of USE using item‐specific response options (see Appendix S1). The 6‐item measure was an adaptation of the 3‐item measure developed by Bradley et al. (2013). The 3‐item measure included questions relating to three aspects of appraisals (externality, agency and threat). For the purpose of this study, we added three additional items reflecting the remaining aspects of appraisals of USE as specified by Brett et al. (2007) – Valence, Abnormality and Controllability. The questionnaire started with a probe (i.e. *Imagine hearing things that other people cannot hear or seeing things that other people cannot see. What would you think about this experience most of the time?*), adapted from the existing measure (Bradley et al., 2013). The probe was followed by six sentences; for each sentence, the young person had to select an ending from five item‐specific options. Item‐specific response options were chosen due to research studies demonstrating the superiority of this approach over the traditional agree/disagree response options (Saris, Revilla, Krosnick, & Shaeffer, 2010). Item responses were sorted beginning with the ones reflecting more negative appraisals of USE and ending with the ones reflecting more positive appraisals of USE. Each response was assigned a numerical value of 1–5 with higher values indicating more positive appraisals.

Before calculating the total appraisals score, a psychometric evaluation of the measure was performed (see Appendix S2). Following the results of this evaluation, participants' responses to items 1, 5 and 6 (Threat, Abnormality, and Valence) were summarised and are subsequently used in main analyses as a measure of participants' appraisals. Participants' responses to remaining three items were included in descriptive analyses only.

##### General Help‐Seeking Questionnaire ‐ GHSQ (Wilson, Deane, Ciarrochi, & Rickwood, 2005)

The GHSQ is 10‐item questionnaire measuring help‐seeking intentions for mental health problems in young people. For each question, the individual reports how likely they are to seek help from a specific source. The total score is calculated by summing participants' responses to all 10 items. The GHSQ has adequate psychometric characteristics when used in general population (Deane, Wilson, & Ciarrochi, 2001; Rickwood, Cavanagh, Curtis, & Sakrouge, 2004). Questionnaire instructions were adapted for the purpose of this study (i.e. asking adolescents about help‐seeking for USE rather than for emotional or personal problems as in the original questionnaire).

#### Secondary outcome measures

##### Fast and Slow Thinking (FaST) questionnaire (Hardy et al., 2020)

FaST is a 10‐item questionnaire measure of reasoning processes. FaST measures fast (e.g. ‘jumping to conclusions’) and slow (e.g. thorough review of the evidence) reasoning biases that can contribute to the development and distress of a wide range of experiences, including USE (Daalman, Sommer, Derks, & Peters, 2013). The original questionnaire was developed for paranoid thoughts, and therefore, the initial probe was changed for this study (i.e. ‘When I have a paranoid or suspicious thought…’ was changed to ‘When I have an upsetting thought about a situation…’) to make the opening more suitable for the general population. The FaST questionnaire has two subscales (Fast thinking and Slow thinking) and adolescent responses to appropriate items were summarised to calculate subscale scores.

##### 11‐item version of the Revised Children's Anxiety and Depression Scale (RCADS) (Radez et al., 2021)

We used the 11‐item version of the RCADS (henceforth referred to as ‘RCADS‐11’), which consists of six items assessing anxiety and five items assessing depression symptoms in young people. In this study, we used an overall total score, which was calculated by summarising participants' responses to all items.

##### Schema Questionnaire for Children – SQC (Stallard & Rayner, 2005)

The SQC is a 15‐item self‐report questionnaire of early maladaptive schemas. Each item is rated on a visual analogue scale of 1–10 ranging from ‘not believing’ to ‘highly believing’ in each statement. Total SQC is calculated by summarising responses to all 15 items and a higher score indicates less adaptive schemas.

### Sample size calculation

We computed a priori power analysis using the ‘WebPower’ package (Zhang & Mai, 2018) in R Studio. With an alpha level of .05, power .80 and effect size (*f*) of .25, the required sample size was approximately *N* = 156 for the planned main analyses (i.e. repeated measures ANOVA).

### Analysis

Questionnaire responses were entered into a spreadsheet, checked and cleaned. We then analysed the missing data and performed sensitivity analyses. This was followed by calculating item‐level descriptive statistics (Means and Standard Deviations) and item‐level differences between the experimental and control group (Mann–Whitney *U*‐tests) for the main outcome measures (the Appraisals Measure and GHSQ). We then calculated baseline scale/subscale differences in the main outcome variables and covariates between the intervention and control group (independent samples *t*‐tests with the effect sizes – Cohen's *d*), and reliability analyses for all included measures. The main analyses included mixed model analyses of variance (ANOVA) with three different time points (pre‐, post‐ and follow‐up) for both main outcome variables (help‐seeking and appraisals of USE) as within subject factors and intervention (USE intervention and control intervention) as between subject factors. For our exploratory hypotheses, covariates (thinking flexibility, dysfunctional schemas and anxiety/depression symptoms) were included in ANOVA models. Prior to running ANOVAs, the data were checked to meet all the assumptions to conduct the analyses. To estimate the practical meaning of our findings, effect sizes (partial η^2^) were calculated. All analyses were conducted in IBM SPSS 27 (IBM Corp., 2020) and we used the α level of .05.

## Results

### Missing data analysis

Across the whole sample, the proportion of missing responses on any of the completed questionnaires was low (range 0–5.2%) (see Appendix S3), and therefore, only complete cases were used in further analyses (Scheffer, 2002). Out of 223 adolescents taking part in the intervention and completing at least one of the first two sets of questionnaires, 34 (15.2%) were lost due to attrition at the follow‐up.

**Figure 1:**
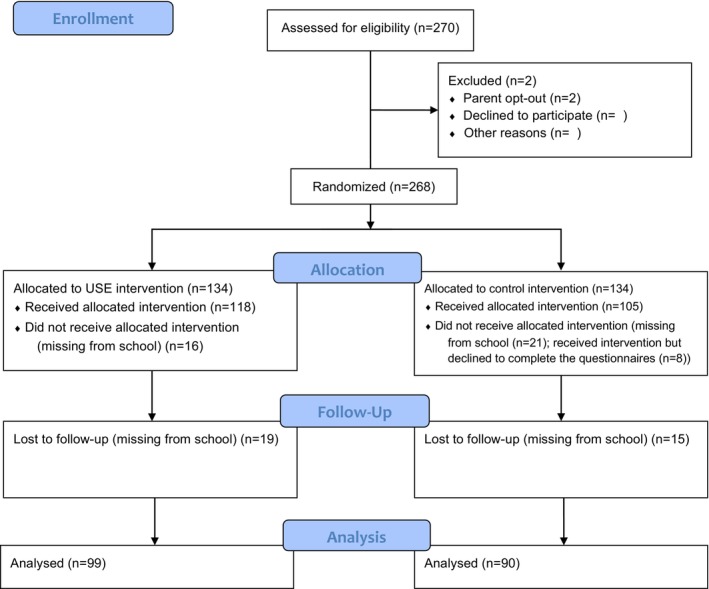
Consort 2010 flow diagram of study phases

We performed sensitivity analyses by conducting a series of *t‐*tests by comparing means of the main outcome variables between adolescents with full questionnaire responses and adolescents that were lost due to attrition (Twisk, 2013). None of the differences were significant, and therefore, it was concluded that the missing data were missing completely at random (MCAR) (Rubin & Little, 2019). Due to the large sample size and adequate power, and the assumption of MCAR being satisfied, the listwise deletion was used to handle the missing data (Kang, 2013). Figure 1 outlines the process of study enrolment.

### Preliminary analyses

Descriptive statistics for individual items included in the main outcome measures across three time points are presented in Appendix S3. Appendix S3 also includes a table of baseline differences in the main outcome measures across both groups. Notably, the only statistically significant baseline difference was observed on GHSQ where participants in the control group reported significantly lower help‐seeking intentions (*t*(205) = 2.29, *p* < .05); however, the effect size of this difference was small (*d* = .32).

### Reliability of measures

Reliability coefficients ranged from .628 to .912. Table outlining reliability coefficients for all measures across the whole dataset and within each group are presented in Appendix S4.

### Main analyses

#### Effect of intervention on adolescents' appraisals of USE

**Figure 2:**
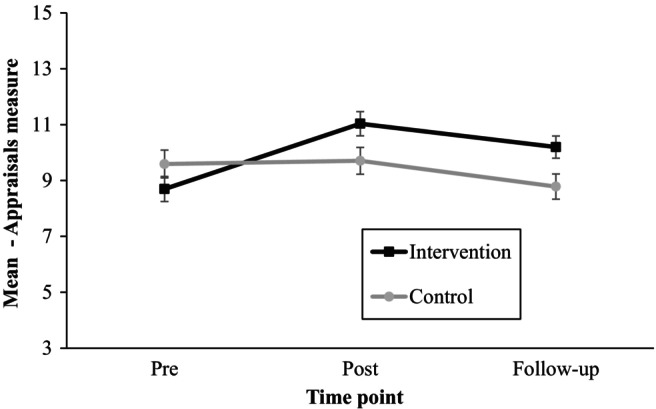
Estimated marginal means for appraisals with 95% error bars for each group and across three different time points

Mixed ANOVA analysis identified a significant effect of time (*F*(1.9, 315.9) = 34.01, *p* < .01, η^2^_P_ = .17), a significant effect of interaction between time and type of intervention (*F*(1.9, 315.9) = 36.41, *p* < .01, η^2^_P_ = .18) and a significant effect of the intervention (*F*(1, 168) = 5.11, *p* < .05, η^2^_P_ = .03) on adolescents' appraisals of USE. Figure 2 illustrates the nature of interaction between the between‐subject variable (intervention type) and within‐subject variable (time).

Postintervention, participants in the USE group reported more positive appraisals of USE compared with those in the control intervention. Post hoc comparisons showed that these differences remained statistically significant at the one‐month follow‐up (*M*_d_ = 1.33, *p* < .01).

#### Effect of intervention on adolescents' help‐seeking intentions for USE

**Figure 3:**
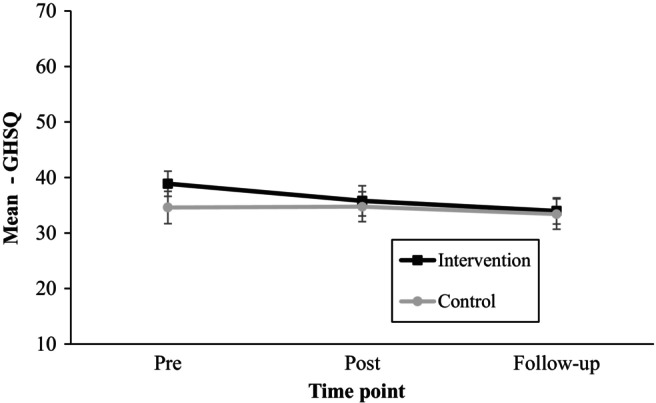
Estimated marginal means for GSHQ with 95% error bars for each group and across three different time points

A significant effect of time on adolescents' help‐seeking intentions for USE was identified (*F*(1.8, 272.7) = 12.04, *p* < .01, η^2^_P_ = .07). There was also a significant interaction between the type of intervention and time (*F*(1.8, 272.7) = 5.30, *p* < .01, η^2^_P_ = .03), indicating differences in help‐seeking intentions between the USE and control group at various time points. However, the effectiveness of intervention itself was not statistically significant (*F*(1, 151) = 1.26, *p* = .26, η^2^_P_ = .01). Further post hoc analyses showed that the interaction was only significant for time point 1 (before the intervention) (*M*_d_ = 4.27, *p* < .05) with the differences between both groups decreasing over time. This can be also seen in Figure 3.

#### Examination of covariates in the relationship between adolescents' appraisals of USE and type of intervention

Correlations between the main outcome variables (Appraisals Measure and GHSQ) and covariates were calculated first (see Appendix S5). Appraisals Measure (across different time points) was significantly negatively associated with the SQC and RCADS‐11. There were no associations between Appraisals Measure and FaST‐FT/FaST‐ST scores, and therefore, these were not included in the model as covariates.

When controlling for SQC and RCADS‐11 scores, the effect of time remained significant (*F*(1.9, 274.1) = 7.34, *p* < .01, η^2^_P_ = .05). Further, the interaction between the type of intervention and time remained significant (*F*(1.9, 315.9) = 28.12, *p* < .01, η^2^_P_ = .16), whereas no significant interaction was identified between SQC score and time (*F*(1.9, 315.9) = .65, *p* = .65, η^2^_P_ = .00) and RCADS‐11 score and time (*F*(1.9, 315.9) = 0.64, *p* = .52, η^2^_P_ = .00).

The main effect of intervention remained significant after controlling for the covariates (*F*(1, 145) = 4.62, *p* < .05, η^2^_P_ = .03), and adolescents' SQC scores were significantly associated with appraisals of USE (*F*(1, 145) = 5.63, *p* < .05, η^2^_P_ = .04), indicating that adolescents with more positive beliefs about themselves/others/the world reported more positive appraisals of USE. Adolescents' RCADS‐11 scores were not significantly associated with the appraisals of USE (*F*(1, 145) = 0.05, *p* = .82, η^2^_P_ = .00).

#### Examination of covariates in the relationship between adolescents' help‐seeking intentions for USE and type of intervention

GHSQ scores were found to be significantly associated with all covariates, and therefore, all four variables were included in a further ANOVA model.

After controlling for covariates, the effect of time on adolescents' help‐seeking intentions was no longer significant (*F*(1.8, 218.9) = 1.32, *p* = .27, η^2^_P_ = .01), but the interaction between the intervention type and time remained significant (*F*(1.8, 218.9) = 4.45, *p* < .05, η^2^_P_ = .03). Controlling for covariates slightly increased the observed effect of intervention of GHSQ scores; however, the effect of intervention itself still failed to reach statistical significance (*F*(1, 125) = 2.78, *p* = .10, η^2^_P_ = .02). Finally, two covariates were identified as significant – FaST‐ST (*F*(1, 125) = 9.42, *p* < .01, η^2^_P_ = .07) and RCADS‐11 (*F*(1, 125) = 10.24, *p* < .01, η^2^_P_ = .08). Results regarding covariates indicate that adolescents with slower thinking and lower level of anxiety and depressive symptoms reported being more likely to seek help for USE.

## Discussion

We set out to investigate the effectiveness of a universal school‐based psychoeducational intervention on adolescents' appraisals of USE and help‐seeking intentions for USE. We also explored the associations between adolescents' schemas, cognitive flexibility and anxiety/depression symptoms and appraisals of USE/help‐seeking for USE. We found that the single‐session intervention was effective in increasing positive appraisals of USE and these effects were sustained over time. Contrary to our expectations, the intervention did not lead to an increase in help‐seeking intentions for USE. Finally, we identified significant associations between (a) adolescents' schemas and appraisals of USE and (b) cognitive flexibility and anxiety/depression symptoms and help‐seeking intentions for USE.

The results of this study are broadly consistent with the previous research investigating the effectiveness of a similar CBT‐based universal school‐based intervention delivered over eight sessions with preadolescent children (CUES‐Ed; Underwood et al., 2021), where researchers reported an overall reduction of cognitive vulnerability (i.e. a composite measure including negative appraisals of USE, as well as stigmatising beliefs about USE and reasoning biases) following the intervention. The researchers reported particularly high reduction in reasoning biases (‘jumping to conclusions’) (Underwood et al., 2021). Although results in our study failed to demonstrate a positive effect of the intervention on adolescents' help‐seeking intentions, these results were not surprising. The intervention was focused primarily on appraisals of common USE (e.g. hearing a noise when falling asleep) emphasising their normality and generally nonthreatening nature, which could lead to the majority of adolescents thinking that help‐seeking for USE is not required. This is consistent with previous research, suggesting that mental health interventions do not necessarily lead to greater help‐seeking intentions but can reduce stigma associated with mental health problems in nonclinical populations (Xu et al., 2018). It is also possible that the opening question of the GHSQ was not well‐phrased for the purpose of this study, as it asks adolescents about general help‐seeking intentions for USE rather than seeking help for *distressing and prolonged* USE. Future research could therefore use a different opening question or include a measure of stigma, which is another psychological process associated with the distress of USE in adult cognitive models (e.g. Garety et al., 2001).

Our study provided some interesting findings regarding the role of covariates in adolescents' appraisals of USE and help‐seeking intentions for USE. In line with previous research (e.g. Anilmis et al., 2015), schemas, reasoning processes, and anxiety and depressive symptoms were identified as significant psychological processes in understanding adolescents' appraisals of USE/help‐seeking intentions for USE. While previous studies have mainly focused on associations between the covariates and negative appraisals of USE (and other PLEs), this study identified covariates associated with positive appraisals of USE and hence, potentially identified young people's *protective* (i.e. rather than risk) factors. These results highlight the opportunity for universal mental health interventions to focus on building up these positives.

Taken together, our study findings suggest that a 40‐min universal school‐based intervention on USE can lead to an increase in adolescents' positive appraisals of USE. These findings, along with the findings of the role of covariates, provide preliminary evidence for applicability of some components of cognitive models (e.g. Garety et al., 2001) in understanding USE in adolescents. Furthermore, these results may have clinical implications by highlighting which psychological processes could be targets of treatment in CBT with adolescents with prolonged and distressing USE. This would need further testing with clinical populations. The results of our study also have other clear research implications by providing grounds for optimism for future school‐based research, which is currently one of the most important topics in adolescent mental health research (see Department of Health & Department of Education, 2017). In particular, brief psychoeducational interventions targeting less functional appraisals may be most cost‐effective, which is particularly important in an educational context, where time and financial pressures are not uncommon. In addition, our identification of significant covariates, such as schemas and anxiety and depressive symptoms, indicates the need for developing approaches that target multiple domains of adolescent mental health and focus on strengthening adolescents' protective factors (Minnard, 2002). This highlights the importance of discussing young people's strengths as a part of the regular school wellbeing curriculum. The CUES‐Ed study provides a good example of a universal school‐based study addressing different psychological factors of mental health in preadolescent children, and future research could focus on developing similar psychoeducational interventions for the adolescent population.

### Limitations

Our study had several limitations. First, all participants were recruited from one school. Furthermore, the school is based in one of the least deprived areas of the UK based on the Index of Multiple Deprivation (IMD; DLUHC, 2019), which limits the generalisability of our findings. Although participants were randomised by class, it is possible that young people from different groups talked to each other about the interventions, meaning that participants' responses at the follow‐up might have been affected by contamination bias. Future research could overcome this issue by recruitment of more schools and cluster randomisation by school. We also did not collect any demographic variables (e.g. gender, ethnicity), which might further explain the observed differences in the main outcome variables at baseline. Further, the final composite score used in main analyses only included three items assessing the perceived threatening aspects of appraisals. A more comprehensive appraisals measure that is developed and validated with young people is required. Although the intervention was well‐accepted by young people, we believe that adolescents' experiences of the intervention could be further explored using qualitative methodology. Finally, our study did not include a measure of distress or impact of USE or a measure of stigma.

## Conclusions

USE are common in adolescence. Appraisals of USE are the main drivers of distress and impact of these experiences, and therefore, interventions aimed at developing more positive appraisals of USE can be helpful. To our knowledge, this is the first study developing and evaluating a universal school‐based mental health intervention targeting adolescents' appraisals of USE. We demonstrated that a one‐session intervention can be effective in improving adolescents' appraisals of USE. The intervention was well‐accepted by young people consulted during the process of the intervention development and by young people taking part in this study. Due to its brevity and easy delivery, this intervention has the potential to be delivered during the regular school curriculum by schoolteachers ensuring wide access and ease of implementation.

## Ethical information

The study was granted a full ethics approval by the Oxford University Research Ethics Committee (CUREC) (reference R79066/RE001). All participants were required to provide an informed consent prior to taking part in the study.

## Supporting information


**Appendix S1.** Appraisals measure.
**Appendix S2.** Psychometric evaluation of the appraisals measure.
**Appendix S3.** Preliminary analyses.
**Appendix S4.** Reliability analysis.
**Appendix S5.** Correlations between the main outcome variables and covariates.
