# Supplementary material for: Understanding unusual sensory experiences: a randomised experimental study of a school‐based intervention for adolescents
Source: Child Adolesc Ment Health. 2023 Mar 23;29(1):14–21. doi: 10.1111/camh.12651 (PMC10877969; doi:10.1111/camh.12651)
Supplement: Supplementary file 1 — Appendix S1. Appraisals measure. Appendix S2. Psychometric evaluation of the appraisals measure. Appendix S3. Preliminary analyses. Appendix S4. Reliability analysis. Appendix S5. Correlations between the main outcome variables and covariates. [file CAMH-29-14-s001.docx]

**Supporting Information -** Understanding unusual sensory experiences: a randomised experimental study of a school-based intervention for adolescents

**Appendix S1 – Appraisals Measure**

*Imagine hearing things that other people cannot hear or seeing things that other people cannot see. What would you think about this experience most of the time?*

1. I would think that that the experience is…[threat]
2. Definitely dangerous to me or other people
3. Slightly dangerous to me or other people
4. Neither dangerous or not dangerous to me or other people
5. Mostly not dangerous to me our other people
6. Definitely not dangerous to me our other people
7. I would think that the experience is caused by something… [externality]
8. Completely outside me (e.g., a real person)
9. Mostly by something outside me
10. Mix by something outside and something inside me
11. Mostly by something inside me
12. Completely by something inside me (e.g. being very tired)
13. I would think that the experience is…[controllability]
14. Completely out of my control
15. Mostly outside my control
16. Partially outside my control and partially under my control
17. Mostly under my control
18. Completely under my control
19. I would think that this experience is...
20. Completely caused by what other people are doing
21. Mostly by what people are doing but some of it caused by other things
22. A mix of both
23. Mostly by other things but some of it caused by what other people are doing
24. Completely caused by other things
25. I would think that… [abnormality]
26. Something is definitely wrong with me
27. Something is probably wrong with me
28. I could be normal or not
29. I am probably normal
30. I am definitely normal
31. I would think that the experience is… [valence]
32. Strongly negative.
33. Somehow negative.
34. Neutral.
35. Somehow positive.
36. Strongly positive.

**Appendix S2 – Psychometric Evaluation of the Appraisals Measure**

A correlation matrix for adolescents’ responses on the Appraisals Measure (across different times) is presented in Table S4.

**Table S1**

*A correlation matrix for items included in the Appraisals Measure*

|  | Appraisal 1 | Appraisal 2 | Appraisal 3 | Appraisal 4 | Appraisal 5 | Appraisal 6 |
| --- | --- | --- | --- | --- | --- | --- |
| Appraisal 1 | 1 | .191** | .096* | .099* | .510** | .433** |
| Appraisal 2 |  | 1 | .125** | .241** | .052 | .071 |
| Appraisal 3 |  |  | 1 | .159** | .183** | .227** |
| Appraisal 4 |  |  |  | 1 | 0.075 | .100* |
| Appraisal 5 |  |  |  |  | 1 | .469** |
| Appraisal 6 |  |  |  |  |  | 1 |

*Note.* **p* < 0.05, ** *p* < 0.01.

Appraisal 1 – 6 = items 1 – 6 of the Appraisals Measure.

Although statistically significant, correlations between most of the items were weak (i.e., < 0.3), and only three relatively strong associations were observed between items measuring Threat, Abnormality and Valence (items 1, 5 and 6). To further examine the dimensionality of the Appraisals Measure, we conducted EFA. Although the Kaiser-Meyer-Olkin (KMO) value was .660 indicating that the correlations between the items were sufficient to conduct EFA, the 6 items did not fit the unidimensional model (χ^2^ = 78.49; *p* < 0.01), indicating that summarising all the item responses and using the sum score in analyses would be inappropriate. Further exploratory analyses indicated that items assessing Externality, Controllability and Agency (items 2, 3 and 4) did not exhibit a consistent pattern of association between themselves or with the remaining three items (1, 5 and 6), and therefore, they were excluded from calculating the Appraisals’ sum score. EFA including only items 1, 5 and 6 identified one factor which explained a high proportion (45.5%) of the variance and with high factor loadings (ranging from 0.61 to 0.72). Participants’ responses to items 1, 5 and 6 (Threat, Abnormality and Valence) were therefore summarised and treated as a measure of participants’ appraisals.

**Appendix S3 - Preliminary Analyses**

**Table S2a**

*Item-level descriptive statistics, between group comparisons*

|  | Whole sample *M(SD)* | | USE | | Control | |
| --- | --- | --- | --- | --- | --- | --- |
|  | *M(SD)* | % missing | *M(SD)* | % missing | *M(SD)* | % missing |
| Appraisal_Pre - Threat | 3.15(1.17) | 0.9% | 3.08(1.14) | 0.9% | 3.24(1.19) | 1.0% |
| Appraisal _Pre - Externality | 3.50(1.14) | 0.5% | 3.50(1.18) | 0.9% | 3.51(1.09) | 0.0% |
| Appraisal _Pre - Controllability | 2.58(1.04) | 0.5% | 2.56(1.00) | 0.9% | 2.59(1.09) | 0.0% |
| Appraisal _Pre – Agency^*^ | 2.91(1.12) | 2.3% | 2.76(1.10) | 3.5% | 3.08(1.13) | 1.0% |
| Appraisal _Pre - Abnormality | 3.03(1.14) | 0.5% | 2.91(1.06) | 0.9% | 3.16(1.21) | 0.0% |
| Appraisal _Pre – Valence | 2.64(0.80) | 0.5% | 2.57(0.80) | 0.9% | 2.71(0.79) | 0.0% |
| GHSQ_Pre - Intimate partner (e.g. boyfriend or girlfriend) | 3.76(1.83) | 3.2% | 3.79(1.68) | 4.4% | 3.74(1.99) | 1.9% |
| GHSQ_Pre - Friend (not related to you) ^*^ | 4.29(1.73) | 0.5% | 4.61(1.61) | 0.9% | 3.93(1.79) | 0.0% |
| GHSQ_Pre – Parent | 5.40(1.93) | 0.5% | 5.56(1.86) | 0.9% | 5.22(1.99) | 0.0% |
| GHSQ_Pre - Other relative/family^*^ member | 3.99(1.80) | 0.5% | 4.24(1.72) | 0.9% | 3.7(1.84) | 0.0% |
| GHSQ_Pre - Mental health professional (e.g., psychologist, social worker, counsellor) | 4.02(2.08) | 0.5% | 4.21(2.05) | 0.9% | 3.80(2.10) | 0.0% |
| GHSQ_Pre - Phone helpline | 2.64(1.73) | 0.9% | 2.75(1.74) | 0.9% | 2.51(1.72) | 1.0% |
| GHSQ_Pre - Doctor/GP | 3.74(2.02) | 1.4% | 3.95(1.99) | 1.7% | 3.50(2.04) | 1.0% |
| GHSQ_Pre - Minister or religious leader (e.g., Priest, Rabbi, Chaplain) | 2.33(1.72) | 1.8% | 2.30(1.63) | 2.6% | 2.36(1.82) | 1.0% |
| GHSQ_Pre - I would not seek help from anyone^*^ | 4.99(2.04) | 0.9% | 5.27(1.90) | 1.7% | 4.69(2.14) | 0.0% |
| GHSQ_Pre - I would seek help for another not listed above (e.g., teacher) | 2.45(1.61) | 2.8% | 2.49(1.58) | 2.6% | 2.4(1.65) | 2.9% |
| Appraisal _Post - Threat^*^ | 3.63(1.12) | 2.3% | 3.93(0.91) | 0.0% | 3.28(1.24) | 5.0% |
| Appraisal _Post - Externality^*^ | 3.50(1.14) | 2.3% | 3.70(1.14) | 0.0% | 3.25(1.10) | 5.0% |
| Appraisal _Post - Controllability | 2.85(1.08) | 2.3% | 2.95(1.15) | 0.0% | 2.73(0.98) | 5.0% |
| Appraisal _Post – Agency | 2.98(1.02) | 3.2% | 2.99(1.01) | 1.7% | 2.96(1.04) | 5.0% |
| Appraisal _Post - Abnormality^*^ | 3.55(1.14) | 2.3% | 3.81(1.07) | 0.0% | 3.23(1.16) | 5.0% |
| Appraisal _Post - Valence^*^ | 2.90(0.80) | 2.8% | 3.03(0.80) | 0.9% | 2.74(0.79) | 5.0% |
| GHSQ_Post - Intimate partner (e.g. boyfriend or girlfriend) | 3.57(1.93) | 5.2% | 3.51(1.87) | 5.4% | 3.64(2.01) | 5.0% |
| GHSQ_Post - Friend (not related to you) | 4.10(1.78) | 2.8% | 4.25(1.79) | 1.7% | 3.93(1.75) | 4.0% |
| GHSQ_Post - Parent | 5.06(2.05) | 2.8% | 4.98(2.09) | 1.7% | 5.14(2.01) | 4.0% |
| GHSQ_Post - Other relative/family member | 3.91(1.93) | 3.2% | 3.92(1.91) | 1.7% | 3.89(1.95) | 5.0% |
| GHSQ _Post- Mental health professional (e.g., psychologist, social worker, counsellor) | 3.62(2.01) | 2.8% | 3.68(2.11) | 1.7% | 3.55(1.90) | 4.0% |
| GHSQ_Post - Phone helpline | 2.51(1.69) | 2.8% | 2.56(1.72) | 1.7% | 2.46(1.67) | 4.0% |
| GHSQ_Post - Doctor/GP | 3.38(2.01) | 3.2% | 3.31(1.95) | 1.7% | 3.45(2.08) | 5.0% |
| GHSQ_Post - Minister or religious leader (e.g., Priest, Rabbi, Chaplain) | 2.31(1.76) | 3.2% | 2.40(1.68) | 1.7% | 2.20(1.85) | 5.0% |
| GHSQ_Post - I would not seek help from anyone | 4.94(2.02) | 3.7% | 4.81(2.00) | 1.7% | 5.09(2.05) | 6.1% |
| GHSQ_Post - I would seek help for another not listed above (e.g., teacher) | 2.52(1.72) | 3.2% | 2.57(1.75) | 2.6% | 2.46(1.70) | 4.0% |
| Appraisal_FU - Threat^*^ | 3.40(1.00) | 0.0% | 3.69(0.97) | 0.0% | 3.09(0.96) | 0.0% |
| Appraisal _FU - Externality | 3.38(0.97) | 0.5% | 3.40(1.08) | 0.0% | 3.36(0.84) | 1.1% |
| Appraisal _FU - Controllability^*^ | 2.61(1.04) | 0.0% | 2.84(1.02) | 0.0% | 2.36(1.02) | 0.0% |
| Appraisal _FU - Agency | 3.07(0.93) | 0.0% | 3.09(0.96) | 0.0% | 3.04(0.90) | 0.0% |
| Appraisal _FU - Abnormality^*^ | 3.29(1.08) | 0.0% | 3.59(1.00) | 0.0% | 2.97(1.07) | 0.0% |
| Appraisal _FU - Valence^*^ | 2.65(0.70) | 4.4% | 2.76(0.71) | 3.1% | 2.53(0.68) | 5.9% |
| GHSQ_FU - Intimate partner (e.g. boyfriend or girlfriend) | 3.52(1.82) | 5.0% | 3.55(1.69) | 4.2% | 3.48(1.96) | 5.9% |
| GHSQ_FU - Friend (not related to you) | 3.98(1.62) | 1.6% | 4.06(1.60) | 0.0% | 3.89(1.65) | 3.4% |
| GHSQ_FU - Parent | 4.93(1.95) | 1.1% | 4.98(1.88) | 0.0% | 4.86(2.03) | 2.3% |
| GHSQ_FU - Other relative/family member | 3.70(1.76) | 2.2% | 3.85(1.76) | 0.0% | 3.53(1.74) | 4.7% |
| GHSQ_FU - Mental health professional (e.g., psychologist, social worker, counsellor) | 3.44(2.03) | 2.2% | 3.42(2.06) | 1.0% | 3.46(2.02) | 3.4% |
| GHSQ_FU - Phone helpline | 2.39(1.63) | 2.2% | 2.31(1.60) | 1.0% | 2.48(1.66) | 3.4% |
| GHSQ_FU - Doctor/GP | 3.24(1.92) | 2.2% | 3.32(2.00) | 0.0% | 3.15(1.83) | 4.7% |
| GHSQ_FU - Minister or religious leader (e.g., Priest, Rabbi, Chaplain) | 2.14(1.56) | 2.7% | 2.22(1.54) | 1.0% | 2.05(1.58) | 4.7% |
| GHSQ_FU - I would not seek help from anyone | 4.38(1.97) | 3.8% | 4.22(1.87) | 3.1% | 4.57(2.08) | 4.7% |
| GHSQ_FU - I would seek help for another not listed above (e.g., teacher) | 2.55(1.68) | 1.6% | 2.56(1.69) | 1.0% | 2.53(1.68) | 2.3% |

*Note.* ^*^ indicates significant (*p* < 0.05) difference in item responses between the USE and Control group (Mann-Whitney U-test).

Pre = before the intervention, Post = immediately after the intervention, FU = 1-month follow-up.

Appraisal = individual item of the Appraisals Measure, GHSQ = General Help-Seeking Questionnaire.

**Table S2b**

*Baseline scores in experimental and control group for all included questionnaire measures*

|  | Whole sample | | USE | | Control | |  |  |
| --- | --- | --- | --- | --- | --- | --- | --- | --- |
| Measure | *M* | *SD* | *M* | *SD* | *M* | *SD* | *t* (*p*-value) | *d* |
| Appraisals Measure | 8.8 | 2.4 | 8.5 | 2.3 | 9.1 | 2.5 | -1.72 (0.09) | -0.23 |
| GHSQ | 37.2 | 11.2 | 38.9 | 10.1 | 35.4 | 12.1 | 2.29 (0.02) | 0.32 |
| FaST-FT | 12.6 | 3.5 | 13.0 | 3.6 | 12.1 | 3.4 | 1.76 (0.08) | 0.24 |
| FaST-ST | 17.1 | 3.7 | 17.2 | 3.4 | 16.9 | 4.0 | 0.51 (0.61) | 0.07 |
| SQC | 59.2 | 19.6 | 60.4 | 19.8 | 57.8 | 19.4 | 0.96 (0.34) | 0.13 |
| RCADS-11 | 11.5 | 6.9 | 11.7 | 6.4 | 11.3 | 7.5 | 0.45 (0.65) | 0.06 |

*Note.* GHSQ = General Help-Seeking Questionnaire, FaST-FT/ST = Fast and Slow Thinking questionnaire – Fast Thinking/Slow Thinking, SQC = Schema Questionnaire for Children, RCADS-11 = 11-item version of the Revised Children’s Anxiety and Depression Scale.

**Appendix S4 - Reliability Analysis**

**Table S3**

*Cronbach’s alpha coefficients for included questionnaire measures*

| Measure | Whole sample | USE | Control |
| --- | --- | --- | --- |
| Appraisals Measure-Pre | 0.637 | 0.628 | 0.636 |
| Appraisal Measure-Post | 0.719 | 0.679 | 0.492 |
| Appraisal Measure-FU | 0.722 | 0.764 | 0.622 |
| GHSQ-Pre | 0.806 | 0.762 | 0.832 |
| GHSQ-Post | 0.822 | 0.828 | 0.815 |
| GHSQ-FU | 0.833 | 0.827 | 0.841 |
| FaST-FT | 0.644 | 0.686 | 0.584 |
| FaST-ST | 0.700 | 0.628 | 0.759 |
| SQC | 0.796 | 0.804 | 0.787 |
| RCADS-11 | 0.891 | 0.868 | 0.912 |

*Note.* Pre = before the intervention, Post = immediately after the intervention, FU = 1-month follow-up.

GHSQ = General Help-Seeking Questionnaire, FaST-FT/ST = Fast and Slow Thinking questionnaire – Fast Thinking/Slow Thinking, SQC = Schema Questionnaire for Children, RCADS-11 = 11-item version of the Revised Children’s Anxiety and Depression Scale.

**Appendix S5 – Correlations between the main outcome variables and covariates**

|  | AM-Pre | AM-Post | AM-FU | GHSQ-Pre | GHSQ-Post | GHSQ-FU | FaST-FT | FaST-ST | SQC | RCADS-11 |
| --- | --- | --- | --- | --- | --- | --- | --- | --- | --- | --- |
| AM-Pre | 1 | .659** | .510** | -0.036 | 0.016 | -0.086 | -0.071 | 0.011 | -.290** | -.226** |
| AM-Post | | 1 | .604** | 0.004 | -0.075 | -.155* | -0.048 | 0.091 | -.286** | -.199** |
| AM-FU |  |  | 1 | -0.117 | -0.113 | -.209** | 0.045 | -0.055 | -0.138 | -0.039 |
| GHSQ-Pre | |  |  | 1 | .861** | .731** | .204** | .336** | -.233** | -.426** |
| GHSQ-Post | |  |  |  | 1 | .741** | 0.115 | .337** | -.254** | -.362** |
| GHSQ-FU |  |  |  |  |  | 1 | 0.128 | .191* | -.221** | -.346** |
| FaST-FT |  |  |  |  |  |  | 1 | .159* | 0.123 | -0.005 |
| FaST-ST |  |  |  |  |  |  |  | 1 | -0.051 | -0.083 |
| SQC |  |  |  |  |  |  |  |  | 1 | .635** |
| RCADS-11 | |  |  |  |  |  |  |  |  | 1 |

**Table S4**

*Pearson’s correlation coefficients between all included measures*

*Note.* **p* < 0.05, ***p* < 0.01. Pre = before the intervention, Post = immediately after the intervention, FU = 1-month follow-up.

AM = Appraisals Measure, GHSQ = General Help-Seeking Questionnaire, FaST-FT/ST = Fast and Slow Thinking questionnaire – Fast Thinking/Slow Thinking, SQC = Schema Questionnaire for Children, RCADS-11 = 11-item version of the Revised Children’s Anxiety and Depression Scale.
